# Bibliometric Analysis of International Scientific Production on Pharmacologic Treatments for SARS-CoV-2/COVID-19 During 2020

**DOI:** 10.3389/fpubh.2021.778203

**Published:** 2022-01-20

**Authors:** Miguel A. Ruiz-Fresneda, Evaristo Jiménez-Contreras, Carlos Ruiz-Fresneda, Rafael Ruiz-Pérez

**Affiliations:** ^1^Department of Crystallography and Structural Biology, Instituto de Química-Física Rocasolano, Consejo Superior de Investigaciones Científicas, Madrid, Spain; ^2^EC3 Research Group, Department of Information and Communication Sciences, University of Granada, Granada, Spain; ^3^EC3metrics Spin-Off, University of Granada, Granada, Spain

**Keywords:** COVID-19, SARS-CoV-2, scientific production, bibliometric analysis, pharmacologic treatments, bibliometric network, visualization

## Abstract

**Background:**

COVID-19 is causing a grave global health and economic crisis and the fight against the pandemic has led to unprecedented scientific activity. Bibliometrics could be a useful tool for guiding future researches lines and promoting international collaboration for an effective treatment. For this purpose, we have conducted a bibliometric analysis of scientific publications on drugs and therapies used to treat COVID-19 during 2020.

**Methods:**

Data source: Web of Science. We gathered data on scientific production relating to drugs used to treat COVID-19. We calculated impact factors and analyzed production by institution, country, and journal, visualizing our results in bibliometric networks.

**Results:**

In 1 year, production relating to COVID-19 exceeded 100 000 publications, with over 6,500 on Drugs and COVID-19. Research into hydroxychloroquine and chloroquine, remdesivir, lopinavir and ritonavir, tocilizumab and convalescent plasma is particularly noteworthy. Mean citations/study range from 11.9 to 15.4. Producer institutions fall into three groups: one in the US and centered on Harvard Medical School; another in Europe led by INSERS; and another in China led by Huazhong University of Science and Technology. Production by journal is widespread but the *Journal of Medical Virology, International Journal of Antimicrobial Agents*, and *American Journal of Transplantation* are noteworthy.

**Conclusions:**

The volume of research that is currently under way is comparable to the magnitude of the pandemic itself. Such a high volume of studies is infrequent and the impact they have achieved has no known precedent. The producing countries are those with highest incidence of the pandemic and greatest scientific potential; moreover, inter-agency and international collaboration has reached extraordinarily high levels.

## Introduction

Coronaviruses are single-stranded RNA genome viruses capable of infecting both humans and animals causing respiratory, gastrointestinal, and neurological illnesses (1, 2). They are characterized by being surrounded by a shell of transmembrane glycoproteins (S proteins), giving them a characteristic morphology in the shape of a crown (3). The virus uses these proteins which anchor themselves to receptors on the host cells they infect.

In the past 20 years, coronaviruses have caused three significant health emergencies, including the current one. In 2002, SARS-CoV (4) was discovered in Guandong, China; it was named thus as it caused a Severe Acute Respiratory Syndrome in patients. It was transmitted from nasal and oral fluids and by physical contact with contaminated surfaces (5). It infected some 8,000 individuals with a mortality rate of 9.5%. In 2012, a new coronavirus with SARS-like symptoms appeared in the Middle East and was named MERS-CoV (*Middle East respiratory syndrome*). This virus is still in circulation and according to the World Health Organization (WHO) has caused 862 deaths with 35% mortality (5). Its transmission rate is lower than that of SARS-CoV with an R_0_ close to 1. Suddenly, in December 2019, a group of patients in Wuhan, China, was diagnosed with pneumonia of unknown origin and a new species of coronavirus, called SARS-CoV-2 due to its similarity to SARS-CoV, was identified. The disease that causes SARS-CoV-2 was named “Coronavirus Disease of 2019” (COVID-19) by the WHO. This new coronavirus has infected an infinitely larger number than its predecessors, with an R_0_ of between 2 and 3.5 (5, 6). Mortality is around 2% (7), and at the time of writing (October 2021) deaths are approaching 5 million worldwide, with more than 240 million diagnosed cases (8). The WHO declared COVID-19 a global pandemic on 11 March 2020.

This pandemic is causing a grave health crisis with serious consequences for the world economy due to the rigorous confinements being imposed. The current situation has led to substantial investment in research funding that has, in turn, triggered the hitherto unprecedented volume of production of studies on SARS-CoV-2/COVID-19. Major publishers and biomedical journals are sharing their content in open access to facilitate the visibility of research as a basis for the generation of new knowledge and the rapid search for solutions (9). A good example is the permanently up-to-date collection of the *International Journal of Epidemiology* > *COVID-19*, which hosts possibly the best series of papers on the incidence and epidemiological characteristics of COVID-19 in different regions and countries around the world. The massive amount of scientific information circulating has led to a rapid response in studies on scientific production related to COVID-19 from both the general (10–18) and socio-economic perspectives (19, 20). Some of these analyse COVID-19 as an unprecedented informational phenomenon and use a wide range of media as their source of data; bibliometric studies, however, specifically use the Web of Science (WoS), Scopus and Medline databases, which are considered more suitable for this type of analysis. Bibliometrics may provide exhaustive evaluations of the most active journals, countries, institutions, or authors in a given research field. For this reason, bibliometric analyses are very important for guiding future research trends and promoting international collaboration between institutions and countries.

Alongside the scientific race against time to develop and administer the first vaccines during 2020, the search for therapeutic and pharmacological treatments for COVID-19 that aim to find palliative remedies for the disease became a priority. Typically, this research is based on repurposing pre-existing drugs, currently prescribed for other pathologies, and now undergoing clinical trials to determine their capacity to prevent the virus from binding to human cells by modifying S proteins or receptor proteins present in cells (3, 21). Other are intended to try to stop replication of the virus, alleviate its inflammatory effects or regulate the disproportionate immune process (cytokine storms) that it triggers. In this line of work, numerous reviews of the research generated have already been published, indicating the speed with which the research has been produced and the need to synthesize the scientific information available (22–27). Furthermore, repositories such as *The COVID-19 Real-Time Learning Network*, sponsored by the Infectious Diseases Society of America (28), are especially useful. For its part, the WHO and agencies such as the Food and Drug Administration (FDA) also update possible applications available for use in COVID-19 (29, 30). However, there is still no controlled scientific evidence for an completely effective treatment. *Science* has recently published a trial of plitidepsin (aplidin) that reports it has an antiviral potency 100 times greater than that of remdesivir (31). At 11 February 2021, another clinical trial has shown that tocilizumab is effective against COVID-19, reducing mortality by 4% in severe infections and, potentially, by 50% when administered with dexamethasone (32). The numerous earlier studies of tocilizumab administration in COVID-19 have been the subject of a meta-analysis (33). Right after COVID-19 was declared a global pandemic and during 2020, the most investigated drugs against COVID-19 included hydroxychloroquine, remdesivir, lopinavir, ritonavir, tocilizumab, azithromycin, among others. After that, some of the drugs listed above along with some new ones including favipiravir, thalidomide, ivermectin, and umifenovir have been considered as the most promising ones during the recent year 2021 (34). Although they are not the goal of the present bibliometrics analysis, it is important to note that vaccines are the most efficient preventive treatment against COVID-19. As a consequence of the rapid development and massive research funding, m-RNA vaccines have been successfully employed for the first time. These m-RNA-based vaccines, such as Pfizer BioNTech and Moderna, consist of messenger RNA (ribonucleic acid) molecules, which contain the genes necessary for viral proteins production, that trigger in an immune response by the host.

The aim of the present study is to analyse international scientific publications through bibliometric indicators on drugs and pharmacological treatments for use in treating COVID-19 during 2020. This work provides novel quantitative data that will be useful for promoting international collaboration between institutions and countries to search for possible clinical responses to the pandemic, as well as for the development of new research.

## Materials and Methods

### Data Source

We have gathered our data from scientific production indexed in the WOS databases (35). This multi-disciplinary international source references the most prestigious scientific publications in the world and is an essential starting point for bibliometric studies providing indicators of production and scientific impact. WOS has been seen to match the current pace of publishing, quickly indexing the special COVID-19 sections that journals have created (*Online articles, Articles in press, Early Access, Latest issue, etc*.) thus enhancing their diffusion and visibility. We found no differences in coverage between WOS and the LitCovid-PUBMED repository. We searched WOS “All Databases,” which includes the biomedical coverage of MEDLINE, BIOSIS and SCIELO. We launched our searches between late December 2020 and January 2021. Given the level of ongoing scientific production, the final data provided in this study may vary, although any differences do not significantly affect our analysis of results.

### Search Strategies and Data Treatment and Analysis

The search strategies used to recover the scientific production indexed in WOS on the subject of study, as well as the treatment and analysis of the data obtained, are described in Supplementary Material.

## Results and Discussion

### Evolution of Scientific Production on Coronavirus

**Figure 1:**
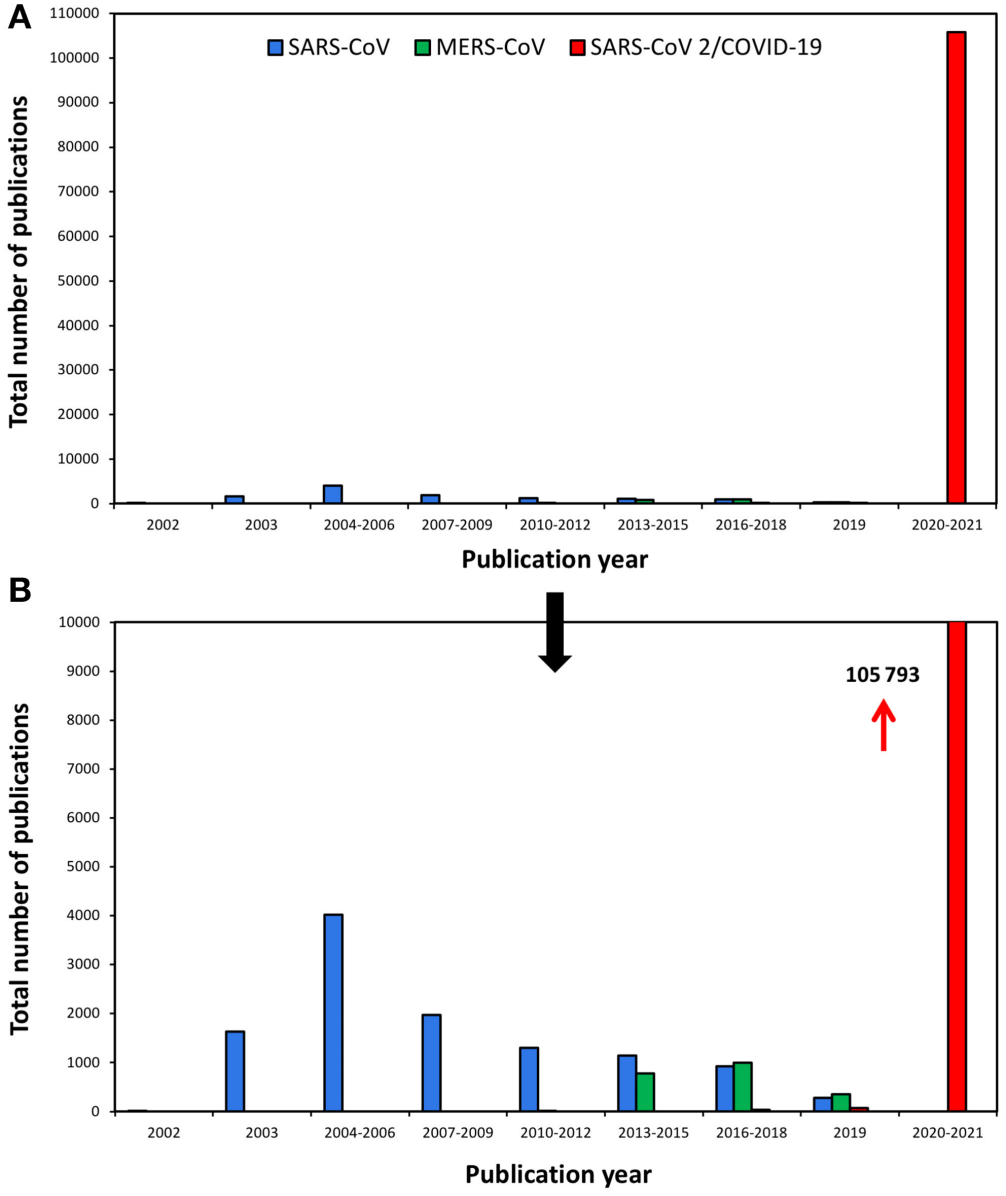
Scientific production on SARS-CoV, MERS-CoV, and SARS-CoV 2/COVID-19 during the last two decades (<2002-2020) represented in two different scales: up to 110000 **(A)** and 10000 publications **(B)**.

Prior to the arrival of SARS-CoV-2/COVID-19, SARS-CoV, and MERS-CoV caused significant worldwide health emergencies that had been studied by the scientific community. Data show that the corresponding scientific production was significant with 1,625 documents dated in 2003 or later (Figure 1)—months after SARS-COV had been detected. Peak production on this coronavirus amounted to 4,018 studies published between 2004 and 2006. Subsequently, production fell to 2,000 documents in 2007–2009; a process that continued until 2017, with 400–500 documents per year. MERS-CoV, identified in 2012, followed a similar evolution but with a smaller volume of studies, corresponding to its lower epidemic potential. Peak production occurred in 2016–2018, with almost 1,000 documents.

However, the upsurge in COVID-19–related production between December 2019 and 2020 has confounded all conceivable predictions of statistical values. In 2020, production rose from almost 0 to over 100,000 documents recorded by WOS, and the number of documents continues to grow. In a single year, all-time production on all coronaviruses has grown seven-fold, reaching around 15,000–20,000 documents. This enormous differences in scientific production between SARS-CoV-2 and other coronaviruses (SARS-CoV and MERS-CoV) can be observed as well analyzing the data obtained in previous works (36, 37).

### Drugs and SARS-CoV-2/COVID-19 Production and Impact

At the end of 2020, scientific production on Drugs and SARS-CoV-2/COVID-19 amounted to 6,533 documents (7% of all SARS-CoV-2/COVID-19 production). Logically, the co-occurrence of these two themes only appears in 2020, when their contents coincided as an object of investigation in the same studies (Table 1). The presence of earlier documents must be attributed to errors in record indexing. The typology of the studies is dominated by articles (60%), followed by the reviews (15%), case reports and clinical trials (10%), early access (10%) and letters (5%). Note that prior to 2020, research on drugs and their repurposing for other pathologies already existed, with substantial annual production rates (Table 1).

**Table 1:** Number of documents on drugs and SARS-CoV-2/COVID-19 (26/12/2020).

**Years**	**≤2013**	**2014**	**2015**	**2016**	**2017**	**2018**	**2019**	**2020**	**Total**
Drugs	1,308,732	66,110	68,380	69,224	70,804	71,186	73,790	60,779	1,789,005
COVID-19	351	26	16	12	5	11	66	105,296	105,793
Drugs and COVID-19	36	3	1	–	–	1	7	6,485	6,533

It is estimated that hundreds of drugs and therapeutic applications are currently being investigated in relation to COVID-19. The present study focuses on those that have received most attention in scientific publications. Table 2 lists the drugs that have led to the production of ≥150 studies, and shows indicators of production and impact measured in terms of citation. In terms of production, hydroxychloroquine—with about 2,000 studies—and chloroquine—with more than 1,000 studies in just 1 year, stand out. These are followed by the antivirals group formed by remdesivir, lopinavir, and ritonavir, with around 1,000 studies produced since February 2020. In terms of impact, both groups have similar values, with between 11.9 and 15.4 mean citations/study (MCS). tocilizumab, convalescent plasma, and azithromycin have led to the production of over 500 studies each.

**Table 2:** Most productive therapies and drugs. Production and impact.

**Drugs/therapies**	**TSP**	**CR**	**MCS**	**CS**	**+CS**	**h-index**
Hydroxychloroquine	1,944	13,205	6.79	5,603	1,147	51
Chloroquine	1,148	13,722	11.95	6,257	1,348	50
Remdesivir	951	11,797	12.4	6,232	1,348	51
Lopinavir	826	12,219	14.79	6,891	1,348	54
Ritonavir	801	12,324	15.39	7,003	1,348	55
Tocilizumab	723	5,648	7.81	5,648	387	7.81
Convalescent plasma	655	5,128	7.83	3,034	473	34
Azithromycin	645	9,468	14.68	6,732	4,227	34
Monoclonal antibodies	584	9,280	15.89	5,465	1,088	45
Angiotensin-converting enzyme 2 receptor	366	7,546	20.62	5,465	1,998	32
Interferon	360	5,779	16.05	4,120	474	32
Dexamethasone	211	470	2.23	406	175	2.23
Methylprednisolone	188	3,573	19.01	2,894	942	23
Total	9,402					

*Production and impact. TSP, Total Studies Produced; CR, citations received; MCS, mean citations/study; CS, citing studies; +CS, citations received by the most cited work; h-index, Number of studies that have received the same or a higher number of citations*.

Numbers of studies related to Methylprednisolone (19.01 MCS) and azithromycin (14.68 MCS) are lower but they have equally impressive impact figures; the latter is the object of study of the single most-cited work recorded with 4,227 citations. Although research into therapeutic treatments with antibodies based on angiotensin-converting enzyme 2 receptors (ACE2) and interferons (INF) began later (April 2020), they have achieved a similar or higher impact (16.05-20.6 MCS) than the previously mentioned drug groups. H-index figures are striking for many of the drugs studied, with some in excess of 50 (total number of works receiving at least 50 citations).

It should be noted that the total sum of documents exceeds the number of documents retrieved (Table 2). This is because numerous studies simultaneously research several drugs and therefore have been counted for each drug studied. The pre-eminent relationships between most–studied drugs are illustrated in the bibliometric network of keyword co-occurrence by article (Supplementary Figure 1). We have identified one pivotal group of articles that centers on hydroxychloroquine and chloroquine, studied in combination with antiviral drugs; another based on tocilizumab; and a third based around convalescent plasma and ACE2.

Finally, it is important to note that several bibliometric analyses about COVID-19 in general terms and vaccines have been already published. However, the present manuscript offers, for the first time, important bibliometric indicators on drugs treatments against COVID-19.

### Producer Institutions, Countries, and Journals

We will now focus on agents of production. Table 3 lists the institutions producing ≥35 studies distributed by drugs. The French INSERS (Institut National de la Santé et de la Recherche Médicale) center leads the list with 213 studies. This multi-center in Health Sciences is considered among the best in the world and ranks alongside the US National Institutes of Health. It stands out for having produced 66 studies on hydroxychloroquine, 41 on chloroquine, and others on remdesivir, lopinavir, and ritonavir. It is followed by the University of Milan, Italy, and Harvard Medical School, which also stand out for studies on hydroxychloroquine and antiviral drugs. With production levels of >100 studies we find Aix-Marseille University, Harvard University, Huazhong University of Science and Technology (Wuhan), the Chinese Academy of Medical Sciences (Beijing) and the Assistance Publique Hôpitaux, Paris, indicating that France and the US are increasingly important producers, as is the People's Republic of China (PRC). The remaining institutions, with more disperse production, underscore the pre-eminence of these countries although many Italian centers appear, as do some in Canada and Iran.

Some differences can be appreciated when analyzing bibliometrics of global scientific research on COVID-19 (38). In COVID-19 general research, Huazhong University of Science and Technology and Wuhan University, where the disease was discovered, are the most producers in terms of publications by far, with 300 and 170 (launched October, 2020) (38). As mentioned above, our results highlights INSERS, the University of Milan, and Harvard Medical School as the top 3 on research on pharmacological treatments against COVID-19 and lead Huazhong University of Science and Technology, and Wuhan University to the 6th and 16th position. This points out the different research specialties among different institutions.

**Figure 2:**
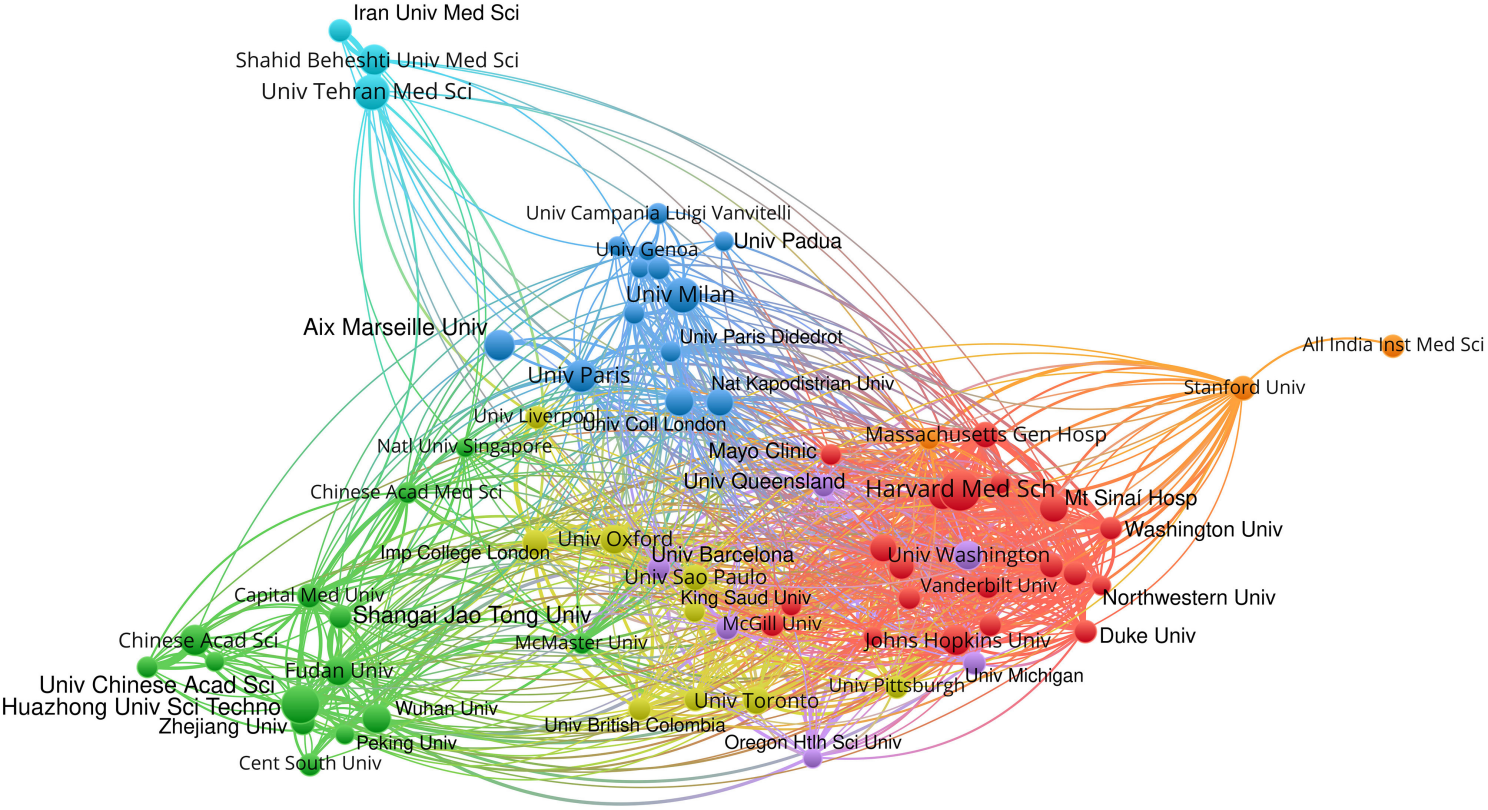
Bibliometric network of production by institution.

Our visualization of the bibliometric network of institutions (co-occurrence ≥ 25) includes the first 68 institutions and depicts three clusters (Figure 2): one located in the US and Canada and centered around Harvard Medical School; another in Europe led by French and Italian universities; and a third in the PRC, with fewer but tightly grouped nodes, centered on Huazhong University of Science and Technology. The US has strong connections indicating tight inter-institutional collaboration; it is closer to the European group and interacts less with the PRC. The PRC cluster shows a marked closeness between actors, although connections are less intense than those observed in the US; three subgroups are evident: one centered on Huazhong University of Science and Technology and connected with Fudan University and Wuhan University; another around the Chinese Academy of Science; and a third around Capital Medical University (Beijing). The Chinese cluster's links to the outside show some interaction with the European cluster through the Chinese Academy of Science and the National University of Singapore. The European cluster shows marked, tight interconnections, especially among the many Italian institutions that interact well with their French counterparts, University College London in the UK, and the National and Kapodistrian University of Athens, Greece. In Europe, we see two peculiarities: on the one hand, some English institutions are absent (University of Oxford and Imperial College London); on the other, INSERM does not appear (ranked 1st in Table 3). This is because the data in Figure 2 give preference to the affiliation of the first signatory, thus reflecting its level of prominence. In contrast, Table 3 shows any institution present among the signatories, and INSERM, although it finances and is involved in a very important part of the work, never appears as the principal institution.

**Table 3:** Number of studies produced by institution and drug (≥35).

**Institutions**	**HY**	**CH**	**RE**	**LO**	**RI**	**TO**	**CP**	**AZ**	**MA**	**AC**	**INF**	**Total**
Inst Nat San et la Recher Medi. Inserm	66	41	16	18	15	12		24	13		8	213
University of Milan	42	20	17	19	20	21		17	14			170
Harvard Med Sch	53	30	27			12		14	14	17		167
Aix Marseille Univ	55	34	12	12	12			29				154
Harvard University	44	25	24			11		13	17	15		149
Huazhong Univ Sci Tech	30	21		18	20	16	20				8	133
Chinese Acad Med Sci	34	27			12	21			28			122
Assistance Publish Hôpitaux Paris APHP	42	19		13	12			19				105
Columbia Univ	29		24			16		14	16			99
Univ Paris	38	27	14					19				98
Univ of Washington	35	38						12		11		96
Chinese Acad Sci			26	19	17		12			17		91
Mayo Clin	31	16	17	11				15				90
University of California System	28	22					14	14		9		87
Johns Hopkins Univ	41	17					17					75
Wuhan Univ	16	20	13	13			12					74
Mcmaster Univ (Ontario)	24	16		15	15							70
Fudan Univ			15	13	13		10		18			69
Icahn Sch Med MT Sinai	26		14				18					58
Stanford Univ	27						15	16				58
Brigham Womens' Hospital	33	22										55
Cent South Univ Changsha, Hunan			14	14	14						12	54
Assis Pub Hôp Marseille	25	16						12				53
New York University	39							13				52
Tehran Univ Med Sci	17				12		13			10		52
Univ Naples Federico II	13			13	13	13						52
Shahid Beheshti Univ Med Sci	19			16	14							49
Univ Hong Kong			13	16	17							46
Univ Campania Luigi Vanvitelli	14			14	15							43
Zhejiang Univ				15	15						13	43
Vanderbilt Univ Nashville, Tenn	23							17				40
Ins de Recherche Pour le Developpement IRD	22	17										39
Capital Med Univ Beijing				12	12					11		35
Sapienza Univ Rome	21					14						35
Total	887	428	246	251	248	136	131	248	120	90	41	2,826

*HY, Hydroxychloroquine; CH, Chloroquine; RE, Remdesivir; LO, Lopinavir; RI, Ritonavir; TO, Tocilizumab; CP, Convalescent Plasma; AZ, Azithromycin; MA, Monoclonal Antibodies; AC, Angiotensin-Converting Enzyme 2 Receptor; INF, Interferon*.

If we look in detail at the collaboration patterns of other centers—such as MacMaster University (Ontario, Canada), Imperial College London, University of Oxford, University of Barcelona, and the University of São Paulo (Brazil)—we see that their positions within the network may be due to the fact that the strong internal interaction of large groups moves them to less well-defined positions. Finally, we would also wish to highlight the peripheral position of Iranian and Indian institutions and the absence of Russian and German institutions, which fail to reach the co-occurrence of collaboration threshold.

**Figure 3:**
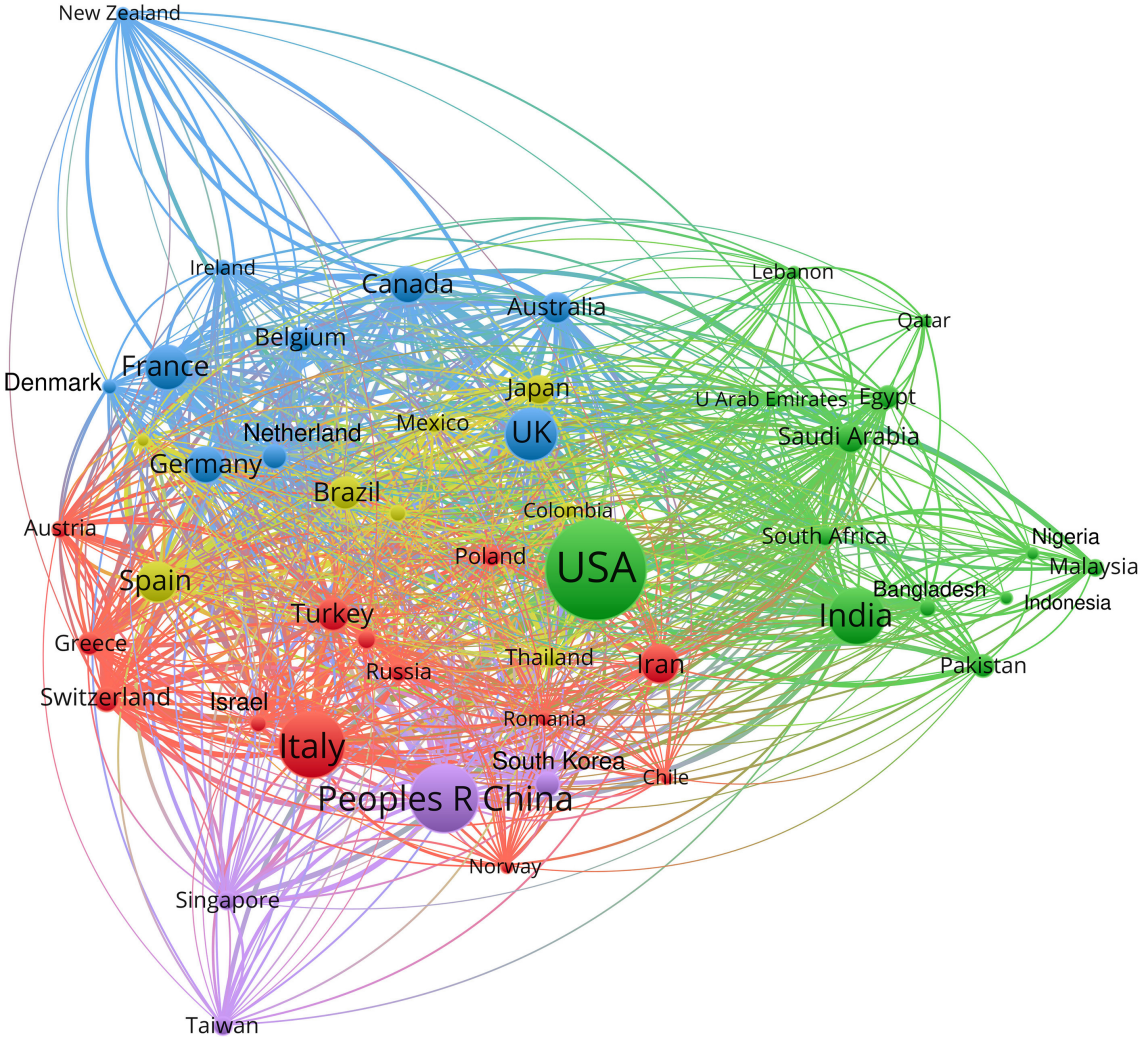
Bibliometric network of production by country.

Our visualization of the bibliometric network by country (Figure 3) allows us to indicate other elements: among the poorly-represented countries we can see those with institutions that have produced > 100 works, which have research potential and have experienced considerable incidence of the pandemic; the positions of the UK, Germany, Canada, Australia and Japan are strengthened or appear for the first time; the positions of Iran and India are strengthened, with significant collaboration in Asia and the Arab world; Saudi Arabia appears; and Spain, Brazil, Belgium, and Turkey are more clearly visible. Bibliometric analysis of global research on COVID-19 highlighted United States, China, Italy United Kingdom, and India as the top countries in number of publications about general research on COVID-19 (38). The research on pharmacological treatments against COVID-19 follows a similar trend, with the same countries in the main positions (Figure 3).

In terms of journal production, the distribution is more disperse. Table 4 shows journals that have published ≥ 40 studies. The *Journal of Medical Virology* [IF (Impact Factor) 2019 = 2.021, Q4, Virology] with 174 papers stands out and is the only journal that has published on all the drugs under study. With more than 100 papers, we find the *International Journal of Antimicrobial Agents* (IF 2019 = 4.62, Q1, Microbiology); and the *International Journal of Infectious Diseases* (IF 2019 = 3.20, Q2, Infectious Diseases). The production of the *American Journal of Transplantation* (IF 2019 = 7.33, Q1, Transplantation) is striking, with 134 studies. The journal, which specializes in organ and tissue transplantation, has created a special section entitled *COVID-19 in Transplantation Infectious Disease*. Their interest in the issue may be due to the fact that transplanted patients are permanently immunocompromised and, therefore, one of the main groups at risk from COVID-19. A similar explanation may justify the production of two journals that have published by far the highest number of papers on a single drug: *Annals of the Rheumatic Diseases* (Rheumatology, IF 2019 = 16.102; Position 2/32) with 63 hydroxychloroquine studies—the drug most frequently researched in the context of COVID-19 and traditionally administered in diseases such as rheumatoid arthritis. *Transfusion* (IF 2019 = 2.80; 40/76, Q3, Hematology) with 61 papers on convalescent plasma; the journal is sponsored by the American Association of Blood Banks and has a panel of experts that issues recommendations for the use of convalescent plasma in COVID-19.

**Table 4:** Production in terms of number of studies by journal and drug (≥40).

**Journals**	**HY**	**CH**	**RE**	**LO**	**RI**	**TO**	**CP**	**AZ**	**MA**	**AC**	**INF**	**Total**
J Med Virol	21	14	13	28	29	27	11	5	10	8	8	174
Am J Transplantation	39		13	15	15	26		16	10			134
Int J Antimicrob AG	37	29	10	8	8	5		21	5	5		128
Int J Infect Dis	30	6		14	12	16	7	19	10			114
New Engl J Med	29		32	21	19							101
Cureus	27	10	14	8	7	10	5	18				99
J Biosol Struct Dynamics	20	14	20	20	14			5		5		98
Ann Rheum Dis	63	7				18			7			95
Trials	30	12		8	9	5	6	9	7		6	92
Am J Trop Med Hyg	29	13	6	13	15			13				89
BMJ (Br Med J)	29		22				17					68
Biorxiv		6	17				14		26	9		72
Medical Hypotheses	17	12		5	6	5		5	6	5	6	67
Transfusion							61					61
Viruses		12	18	6	10				14			60
JAMA		7	18			5	22	7				59
Lancet		5	19	7	7	12						50
Front Pharmacol	11	6	7	5		6	6				8	49
Clin Infect Dis	17		10			7	6					40
Eur Rev Med Pharmacol Sci	10	5	5	9	9				6			44
Front Immunol						10	10		13		10	43
J Antimicrob Chemother	11			15	17							43
Eur J Pharmacol		11	9	8	8			5				41
Nature		5	11						20	5		41
Pharmacol Res		8	8	10	9						6	41
Ann Intern Med	17	12	5					6				40

*HY, Hydroxychloroquine; CH, Chloroquine; RE, Remdesivir; LO, Lopinavir; RI, Ritonavir; TO, Tocilizumab; CP, Convalescent Plasma; AZ, Azithromycin; MA, Monoclonal antibodies; AC, Angiotensin-converting enzyme 2 Receptor; INF, Interferon*.

Finally, given the clinical condition presented by patients with COVID-19, the presence of Medicine, General, and Internal journals at the top of the rankings is logical. Here we highlight the presence of the *New England Journal of Medicine* (IF 2019 = 74.69, Q1, first in its speciality, 1/65) with 100 papers; and the *Lancet* (IF 2019 = 60.39, Q1, 2/65), with 50 studies, but ranking in an intermediate position. The remaining journals appear in a long list included publications in the field of Pharmacology and Pharmacy, and dominated by those in Microbiology, Infectious Diseases, and Immunology.

## Conclusions

The present study clearly demonstrates that global research on drugs and pharmacological treatments against COVID-19 has been massive and has reached unprecedented levels in terms of number of publications, citations, impact factor, and cooperation between countries and institutions. Our results showed that the therapy against COVID-19 has been focused on the efficiency of existing medical treatments for their application to this disease. The studies and citations they have received during a single year (2020) have reached unprecedented levels (14–20 MCS for azithromycin and ACE2, for example) in comparison with the most prestigious journals, i.e., *Nature* or *Science* years before (8.4 and 9.6 MCS during in 2018–2019, respectively). Similarly, h-index scores have reached equally unprecedented values higher than 50 in 2020, while the highest scores of articles in *Nature* or *Science* only reached 40. However, it should be noted that to a large extent, the aforementioned citation activity feeds back into itself with 14–32% of self-citation between studies, a figure higher than that typical in periods of normal scientific activity. Producer countries and institutions correlate with three variables: the origin of SARS-CoV-2; the countries most affected by the pandemic; and world leaders in research. The institutions are also those that conduct research into the most promising drugs, including the antimalarials hydroxychloroquine and chloroquine; antiviral drugs remdesivir, lopinavir and ritonavir; the antibiotic azithromycin; tocilizumab, and convalescent plasma. Collaboration between institutions and between countries is significant, and transparency and the exchange of research results has certainly led to rapid progress in the fight against the disease and clinical treatments have been approved. Substantial inter-institutional research has taken place between centers in the same country and between countries in the same geographical area.

The results here provided can be very useful for the development of new and existing researches lines on pharmacological treatments against COVID-19, as well as to promote international collaboration, which in turn would help to achieve an effective cure against this horrible disease.

## Author Contributions

MAR-F and RR-P: conceptualization, formal analysis, and investigation. EJ-C, CR-F, and RR-P: methodology. MAR-F, EJ-C, and RR-P: software and writing—original draft preparation. MAR-F, CR-F, and RR-P: writing—review and editing. MAR-F: visualization. RR-P: supervision and project administration. RR-P and EJ-C: funding acquisition. All authors contributed to the article and approved the submitted version.

## Funding

This work was supported by the PAIDI (Plan Andaluz de Investigación, Desarrollo e Innovación 2020) program (Junta de Andalucía. Spain. HUM.777-EC3 Research Group).

## Conflict of Interest

The authors declare that the research was conducted in the absence of any commercial or financial relationships that could be construed as a potential conflict of interest.

## Publisher's Note

All claims expressed in this article are solely those of the authors and do not necessarily represent those of their affiliated organizations, or those of the publisher, the editors and the reviewers. Any product that may be evaluated in this article, or claim that may be made by its manufacturer, is not guaranteed or endorsed by the publisher.
