# Supplementary material for: Bibliometric Analysis of International Scientific Production on Pharmacologic Treatments for SARS-CoV-2/COVID-19 During 2020
Source: Front Public Health. 2022 Jan 20;9:778203. doi: 10.3389/fpubh.2021.778203 (PMC8811030; doi:10.3389/fpubh.2021.778203)
Supplement: Supplementary file 1 [file Data_Sheet_1.docx]

Supplementary Material

# Materials and methods

## Search strategies

To gather data comparing scientific production on previous coronaviruses and that on SARS-CoV-2, we conducted searches in WOS *“All Database”>Advanced Search>TS=Topic*. For SARS-CoV, the **#1** search was performed using the equation: *TS=("SARS-CoV" OR "SARS CoV" OR “SARS-CoV-1” OR “SARS CoV-1” OR "Severe Acute Respiratory Syndrome"). Timespan=1900-2019.*  For MERS-COV, the **#2** search used the equation: *TS=(“MERS CoV” OR “MERS-CoV" OR "Middle East Respiratory Syndrome"). Timespan=1900-2019.* For SARS-CoV-2 / COVID-19, the **#3** search was more complex, using the equation: *TS=(“Wuhan coronavirus” OR “Wuhan seafood market pneumonia virus” OR “Covid19*” OR “Covid-19*” OR “Covid-2019*” OR “coronavirus disease 2019” OR “SARS-CoV-2” OR “Sars2” OR “2019-nCoV” OR “2019 novel coronavirus” OR “Severe Acute Respiratory Syndrome Coronavirus 2” OR “2019 novel coronavirus infection” OR “coronavirus disease 2019” OR “coronavirus disease-19” OR “SARS-CoV 2019” OR “SARS-CoV-19”). TimeSpan=All years.*

To achieve our principal objective, we designed a search to identify within the set of documents retrieved in **#3**, those that studied pharmacological treatments and therapeutic agents. Our aim was to find the point of intersection between SARS-CoV-2/COVID-19 and the names of these agents and drugs. To do so, we designed the **#4** search by using a list of terms that identify these drugs and agents. We chose those drugs and agents analysed in the abovementioned reviews as an initial list. This was supplemented by an analysis of repositories, such as *The COVID-19 Real-Time Learning Network*> *Therapeutics & Interventions;^27^* other drugs and therapeutic groups included in the (not always well structured) WHO and FDA classifications were also added. We also located drug effectiveness monitoring for COVID-19.^34^ These sources usually group studies under the name of each drug or treatment and within the therapeutic group to which they belong. As a result, the **#4** search was constructed using the equation: *TS=(“ACE-2 Recombinant” OR “Amodiaquine” OR “Anakinra” OR “Angiotensin-Converting Enzyme 2 Receptor” OR “Antibiotic and Antifungal Agent” OR “Anticoagulants” OR “Anticytokine” OR ”Immunomodulatory” OR “Arbidol” OR “Ariptadil” OR “Azithromycin” OR “Bariatinibib” OR “Baricitinib” OR “BDB-001 injection” OR “Beclomethasone” OR “Betamethasone” OR “Bevacizumab Avastin” OR “Camostat mesilate/nafamostad” OR “Captopril” OR “Cardiovascular Drugs” OR “****CAStem cell injection” OR “****Chloroquine” OR “Chlorpromazine” OR “Clomipramine” OR “Colchicine” OR “Convalescent Plasma” OR “Corticosteroids” OR “Cyclosporin A” OR “Cytosorb” OR “Darunavir” OR “Dasatinib” OR “Dexamethasone” OR “Dexmedetodine” OR “Disulfiram” OR “Eculizumab” OR “EIDD-2801” OR “Enoxaparin” OR “Enalapril” OR “Favipiravir” OR “Gemcitabine” OR “Glucocorticoid” OR “Human C5 monoclonal antibody” OR “Hydrochloride” OR “Hydroxychloroquine” OR “Imatinib mesylate” OR “Invermectin” OR “Immunoglobulin Therapy” OR “Indomethacin” OR “Interferon Alfa-2B” OR “Interferon Beta-1B” OR “Leflunomide” OR “Loperamide” OR “Lopinavir” OR “Mefloquine” OR “Melatonin” OR “Metformin” OR “Methylprednisolone” OR “Molnupiravir” OR “Monoclonal antibodies” OR “Naproxen” OR “Niclosamide and Ivermectin” OR “Nitazoxanide” OR “Nitazoxanide and Tizoxanide” OR “Nitric Oxide and Epoprostenol” OR “Oleandrin” OR “Oseltamivir” OR “Oseltamivir and intravenous Peramivir” OR “Prednisolone” OR “Prednisone” OR ”Promethazine” OR “Remdesivir” OR “Ribavirin” OR “Ritonavir” OR “Ruxolitinib” OR “Sarilumab” OR “Siltuximab” OR “Sirolimus” OR “Sivelestat sodium” OR “Stem Cell Therapy” OR “Tamoxifen” OR “Teicoplanin” OR “Terconazole” OR “Thiazolidinediones” OR “Tocilizumab” OR “Toremifene” OR “Umifenovir”).*

Finally, the intersection between **#3** and **#4** was constructed by using “Combine **#3 AND #4**”. Our **#3** search returned 105 793 documents and **#4** found 1 789 005. Combine **#3 AND #4** returned 6533 documents on Drugs & SARS-CoV-2/COVID-19.

## Data processing and analysis

The data obtained from searches **#1**, **#2**, **#3** were tabulated and we produced a graph of the evolution of scientific production relating to the different coronaviruses between 2002 and 2020. The 6533 complete bibliographic records resulting from the search “Combine **#3 AND #4**” were processed and standardized in Excel. We designed a database to analyse the production and impact of the studies recorded and study investigation into the most frequently researched drugs disseminated by journals, institutions and producer countries. To visualize the bibliometric networks, we used VOS-viewer software (https://www.vosviewer.com/), which works with units of analysis (authors, organizations, etc.) and of measurement (links, frequency, centrality, distance), to illustrate our results by grouping similarities in clusters. All documents were previously debugged using bibexcel (https://homepage.univie.ac.at/juan.gorraiz/bibexcel/), which enabled us to unify term entries. To build the co-occurrence networks we generated vectors, which were pre-displayed in PAJEK (<http://mrvar.fdv.uni-lj.si/pajek/>), with definitive drawings created in VOS-viewer. We used this process because VOS-viewer is limited in that it labels nodes based on an internal, non-modifiable schedule. We labelled as many nodes as possible while guaranteeing the set were correctly displayed.

# Supplementary Figures


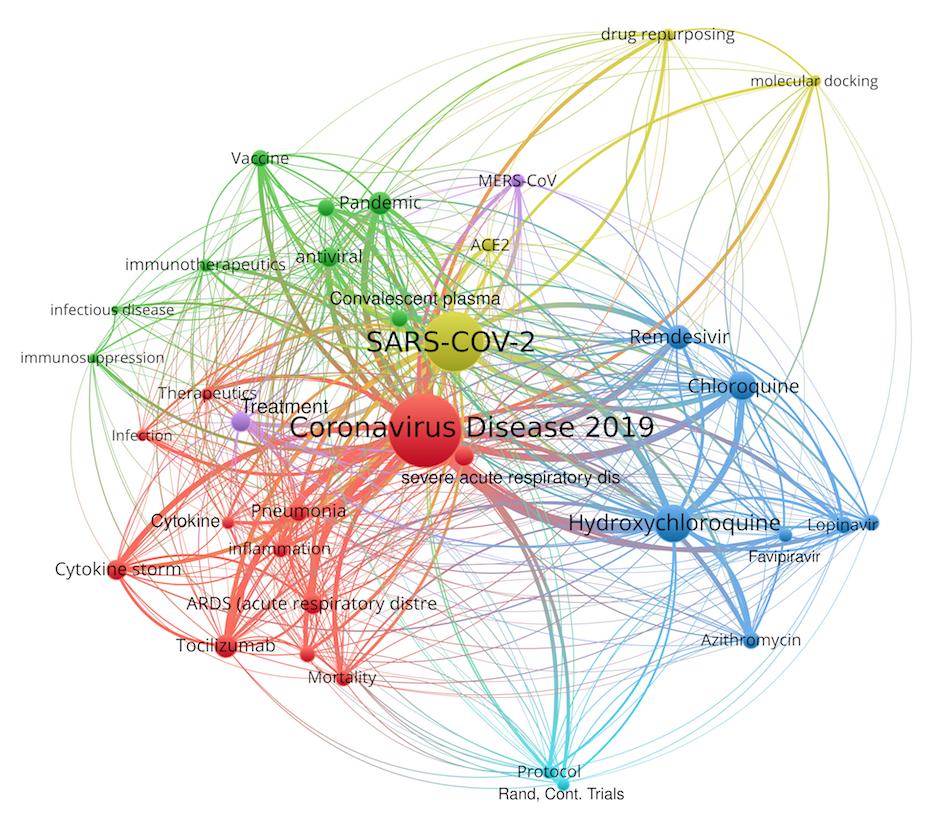


**Supplementary Figure 1**. Bibliometric network for the co-occurrence of article keywords
